# The Higher Response of Vascular Endothelial Growth Factor and Angiotensin-II to Human Chorionic Gonadotropin in Women with Polycystic Ovary Syndrome

**DOI:** 10.22074/ijfs.2015.4176

**Published:** 2015-02-07

**Authors:** Junwei Qu, Yena Che, Pei Xu, Yanjie Xia, Xiaoke Wu, Yong Wang

**Affiliations:** 1 Department of Gynecological Oncology Surgery, Jiangsu Cancer Hospital and Institute, Nanjing 210009, China; 2 Translational Medicine Center and Jiangsu Key Laboratory of Molecular Medicine, Medical School of Nanjing University, Nanjing 210093, China; 3 Department of Obstetrics and Gynecology, The First Affiliated Hospital, Heilongjiang University of Chinese Medicine, Harbin 150040, China

**Keywords:** Polycystic Ovary Syndrome, Vascular Endothelial Growth Factor, Angiotensin-II, Ovarian Hyperstimulation Syndrome

## Abstract

**Background:**

This research investigated the response of vascular active factors, vascular endothelial growth factor (VEGF) and angiotensin-II (AT-II) to ovarian stimulation during 24 hours in patients with polycystic ovary syndrome (PCOS).

**Materials and Methods:**

In this clinical trial study, 52 patients with PCOS and 8 control cases were stimulated with human chorionic gonadotropin (HCG) on the 4^th^ to 7^th^ day of the patients’ natural or induced menstrual cycles. We measured VEGF and AT-II by radioimmunoassay before the injection (0 hour) and 3, 8, 12, 18 and 24 hours after the stimulation.

**Results:**

After ovarian stimulation, there was substantially higher level of VEGF in typical PCOS patients than the other three groups at the 3 hour time point (p<0.05), while there were no significant differences in VEGF at all the other time points among the four groups. As for AT-II, before and at all time points after the ovarian stimulation, it seemed that the AT-II levels in patients’ sera with different phenotypes of PCOS by the Rotterdam criteria were all higher than in the control group although the differences were not statistically significant. The level of AT-II in typical PCOS patients was also significantly higher than the other three groups at the 3 hour time point (p<0.05), while no significant differences at all the other time points among the four groups were observed.

**Conclusion:**

The response to the stimulation varied among patients with different phenotypes of PCOS according to the Rotterdam criteria. Serum VEGF and AT-II were possible contributors to an increased risk of developing ovarian hyperstimulation syndrome (OHSS) in patients with typical PCOS during the early follicular phase (3 hours) after ovarian stimulation (Registration Number: NCT02265861).

## Introduction

Polycystic ovary syndrome (PCOS) is a complex and common gynecological endocrine disorder that occurs in 5-10% of reproductive women (1). In China, this disorder is found in 50-60% of outpatients in gynecologic endocrinopathy clinics (2). It is characterized by a polycystic ovary, ovarian hyperandrogenism (HA), and anovulation although there are several different phenotypes of PCOS according to the Rotterdam criteria (3). A key pathophysiological feature of PCOS is an increase in ovarian mass caused by new blood vessel proliferation in the stroma and theca interna (4). Ultrasonographic assessment of the stromal area and blood flow is currently used as a diagnostic test (5). In ovarian hyperstimulation syndrome (OHSS), massive ovarian enlargement has been reported with PCOS as one of its risk factors (6). Vascular active factors, such as vascular endothelial growth factor (VEGF), angiotensin-II (AT-II), insulin-like growth factor-1 (IGF-1), and some cytokines may be involved which have certain relevance for PCOS pathophysiology (7). Ovarian stimulation may change the production of vascular active factors and different phenotypes of PCOS may have different responses to the stimulation.

VEGF is a 46 kd dimeric protein. Its expression is increased where vascular proliferation is active. The most important sources of VEGF in the female reproductive system are local macrophages and granulosa cells, and the production of VEGF can be increased by human chorionic gonadotropin (HCG) (8). VEGF is one of the most likely candidates for promoting angiogenesis in PCOS and OHSS. The serum VEGF level is increased both in the patients with PCOS and OHSS (9). The increased number of actively secreting granulosa lutein cells and the increased secretory capacity of each granulosa cell both contribute to excessive VEGF production (10).

In PCOS, the Renin-Angiotensin System (RAS) is accentuated. The roles of AT-II have been proposed in growth and atresia of follicles, oocyte maturation, ovulation, corpus luteum formation, steroidogenesis, and corpus luteum regression (11). The level of AT-II in peripheral blood is higher in PCOS patients than in non-PCOS women. AT-II concentration is positively correlated with the level of testosterone (T) in PCOS patients (12). Therefore, components of the RAS, such as AT-II, may be involved in PCOS pathophysiology.

VEGF and AT-II belong to different molecular systems, however both can act as vascular active factors. The aim of this study is to investigate whether ovarian stimulation increases serum vascular active factors such as VEGF and AT-II within 24 hours after stimulation in patients with PCOS during the early follicular phase.

## Materials and Methods

This clinical trial study enrolled women with or without PCOS who entered an *in vitro* fertilization (IVF) program at the hospital affiliated to the Medical School of Nanjing University. This research was approved by the Medical Ethics Committee of the school and informed consent was obtained from the participating subjects. A total of 60 women were recruited and divided into four groups by the Rotterdam criteria according to three typical characteristics: 1. biochemical characteristics of HA, 2. chronic anovulation, and 3. polycystic ovarian morphology (PCO). Group 1 (typical PCOS) was composed of 21 women who had all three of the above features, group 2 (PCOS without PCO) included 14 women with both biochemical characteristics of HA and chronic anovulation, group 3 (PCOS without HA) included 17 women with both chronic anovulation and PCO, and group 4 consisted of 8 volunteers without any biochemical characteristics of HA, chronic anovulation or PCO. This group served as the controls for the three PCOS groups. No patients were prescribed any hormonal prescriptions during the three months preceding the study. The clinical manifestations and the basic data are shown in table 1.

An ovarian stimulation test, which mimicked the common ovulation protocol (10) was performed by the administration of a single intramuscular (i.m.) injection of HCG (5000 IU, The First Biochemical Pharmapeutic, Shanghai, China) during the early follicular phase of the same menstrual cycle (4^th^ to 7^th^ day of the cycle).

Blood samples from all subjects were collected immediately prior to the injection (H0), and at 3 (H1), 8 (H2), 12 (H3), 18 (H4), and 24 (H5) hours after the injection. The serum was stored at -80˚C until further analyses of VEGF and AT-II levels by immunoradioassay. Concentrations of androstenedione (A), T and estradiol (E_2_) at H0 were also measured.

**Table 1:** Basic clinical and hormonal profiles in the four groups


	Group 1	Group 2	Group 3	Group 4

**N**	21	14	17	8
**Age (Y)**	30.1± 4.03	30.5± 3.69	31.0 ± 4.12	29.5 ± 5.12
**BMI (kg/m^2^)**	24.5± 1.29	23.5± 0.8	22.3 ± 0.99	22.4 ± 0.76
**WHR**	0.82± 0.02	0.80± 0.02	0.80 ± 0.02	0.78 ± 0.02
**T (nmol/L)**	2.09± 0.14 ^a, b^	2.09± 0.12 ^a , b^	1.22 ± 0.13	0.73 ± 0.14
**FTI**	10.76 ± 1.57 ^a, b^	6.44± 0.7	3.52 ± 0.62	1.58 ± 0.48
**A (nmol/L)**	9.56± 0.63 ^a, b^	8.07± 0.8 ^a^	6.97 ± 0.62	4.47 ± 0.36
**E_2_ (pg/ml)**	34.9± 5.09	37.5± 5.48	29.2 ± 2.57	30.8 ± 3.26
**E_2_/T**	17.8± 2.73^a^	18.8± 5.76^a^	33.9 ± 8.15	78.0 ± 37.8


Note: A total of 60 women were recruited and divided into four groups by the Rotterdam criteria. a; P<0.05 vs. group 4, b; P<0.05 vs. group 3, BMI; Body mass index, WHR; Waist to hip ratio, T; Testosterone, FTI; Free testosterone index, A; Androstenedione and E_2_: Estradiol. Among the groups there were no differences in age, BMI and WHR. The T, A, and E_2_ levels of the polycystic ovary syndrome (PCOS) groups were significantly higher than the control group.

### Statistical analysis

Tests were two-sided and conducted at α <0.05. In general, the methods for the multiple comparisons were based on analysis of variance (ANOVA) and analysis of covariance (ANCOVA) to identify group differences with the Bonferroni correction when appropriate. Logarithmic transformation of data was performed for data that were not normally distributed. For all analyses, a two-tailed p<0.05 was considered statistically significant. Unless otherwise noted, all results were described as mean ± SD. Correlations were examined by linear regression analysis. Statistical analysis was performed using the SPSS 11.0 software package for Windows.

## Results

The subject characteristics of the four groups are summarized in table 1. Among the groups there were no differences in age, body mass index (BMI) and waist to hip ratio (WHR). There were significantly higher T, A, and E_2_ levels of the PCOS groups compared to the control group (Table 1).

**Fig 1:**
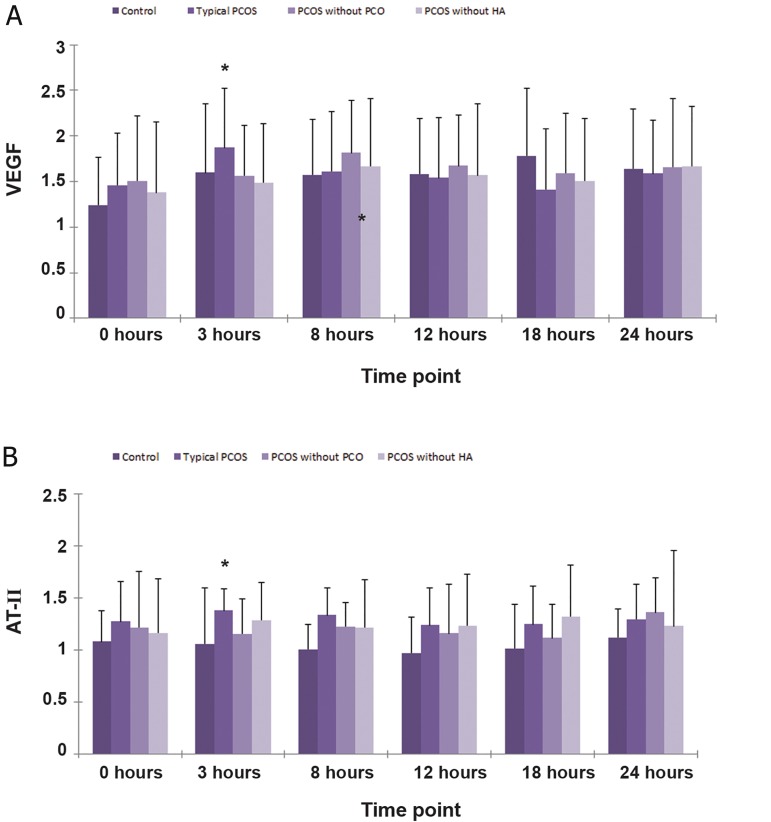
Vascular endothelial growth factor (VEGF) (A) and angiotensin II (AT- II) (B) response to stimuli during the ovarian stimulation test. The level of VEGF and AT- II in typical polycystic ovary syndrome (PCOS) patients (group 1) was substantially higher than the other three groups at the 3 hour time point. Note: Logarithmic transformation of data was performed for measures that were not normally distributed. All results are expressed as Log^10^ (x ± s). *; P<0.05, PCO; Polycystic ovarian morphology and HA; Hyperandrogenism.

Before ovarian stimulation, there were no significant differences in VEGF between the four groups (Fig 1A). After ovarian stimulation, the level of VEGF in the typical PCOS patients (Group 1) was substantially higher than the other three groups at the 3 hour time point (p<0.05), while there were no significant differences in VEGF at the other time points among the four groups (Fig 1A). As for AT-II, before and at all time points after ovarian stimulation, it appeared that AT-II levels in the patients’ sera with different phenotypes of PCOS according to the Rotterdam criteria were all higher than in the control group (Fig 1B), however these differences were not statistically significant. The level of AT-II in typical PCOS patients (Group 1) was also significantly higher than the other three groups at the 3 hour time point (p<0.05), while no significant differences at all the other time points among the four groups (Fig 1B) were observed.

## Discussion

Abnormal expression of VEGF, accompanied by abnormal angiogenesis and vascular permeability has been suggested as a cause of several diseases including PCOS and OHSS (13-16). Increased secretion of VEGF in the serum of patients with PCOS may be induced as a result of an increased number of actively secreting granulosa lutein cells that have both increased secretory capacity and upregulated gene expression level (17). The expression level of VEGF is increased in the hyper-echogenic stroma of PCOS and it induces subsequent stroma growth by promoting microvascular permeability (18, 19). Androgen is secreted in the theca interna and the latter will grow abnormally where vascular proliferation is active. VEGF can affect vascular endothelial cell proliferation and vascular permeability (20). PCOS is one of the risk factors of OHSS (21). The significance of VEGF is its contribution to the induction and progression of OHSS during ovarian induction. VEGF has also been suggested to be responsible for OHSS which is an iatrogenic and potentially life-threatening complication of ovulation induction for the treatment of infertility. Our findings have demonstrated that ovarian stimulation increased VEGF expression during the early follicular phase of typical PCOS patients, which indicated that VEGF might be directly involved in OHSS pathogenesis (22).

Despite the fact that OHSS occurs in the luteal phase after ovarian induction, we have proposed that VEGF should be monitored during the early follicular phase. However, in this study, the level of VEGF in the PCOS without PCO or HA did not significantly increase. This phenomenon has demonstrated that the responsiveness of patients with typical PCOS to ovarian stimulation was greater than that of the PCOS without PCO or HA. As we know, women of both typical PCOS and PCOS without HA are at higher risks for OHSS (23). However according to our observation, the VEGF response patterns in these two groups differed; group 1 was more sensitive than group 3. This phenomenon may suggest that the typical PCOS is more severe than the phenotypic PCOS. Further research is needed to clarify this phenomenon.

AT-II plays an important role in RAS and it affects the reproduction cycle at different stages (24). Disturbances in ovarian RAS can be the cause or the result of such reproductive disorders as PCOS and OHSS. Our data have demonstrated that ovarian stimulation led to AT-II expression in patients with typical PCOS. Consistent with a previous report, AT-II might play an important role in PCOS pathophysiology (25). AT-II has been shown to modulate the local functions of ovaries, such as ovarian steroidogenesis and formation of the corpus luteum, in addition to stimulation of oocyte maturation and ovulation via AT-II receptors on granulosa cells (26).

In addition to the circulating RAS, the ovary has been recently demonstrated to exhibit its own RAS products and activities. Such an intrinsic RAS can modulate the local functions of ovaries such as follicular development, ovulation, and formation of the corpus luteum (27). As AT-II is mainly produced by follicular theca cells and granulosa lutein cells (28), patients with PCOS have a higher risk of developing OHSS because of exogenous gonadotropin for ovulation induction. Results of these studies indicate that RAS is more active in the ovaries of PCOS patients.

Although OHSS occurs during the luteal phases, the current study was performed during the follicular phase. Ovarian stimulation increased the AT-II levels during the follicular phase in PCOS patients. These results, taken together with other parallel studies (29), led us to consider serum AT-II as a possible contributor to a greater risk of OHSS in patients with PCOS during ovulation induction. Inhibition of RAS, commensurate with the changes of serum AT-II concentrations, might be used as a therapeutic approach.

## Conclusion

In conclusion, the response of the ovaries to HCG stimulation differs in patients with different phenotypes of PCOS according to the Rotterdam criteria. The typical PCOS is more severe than the phenotypic PCOS. Further research is needed to clarify this phenomenon. Serum VEGF and AT-II levels may be considered as biomarkers to predict risks of developing OHSS in patients with typical PCOS during the early follicular phase, at 3 hours after ovarian stimulation.
